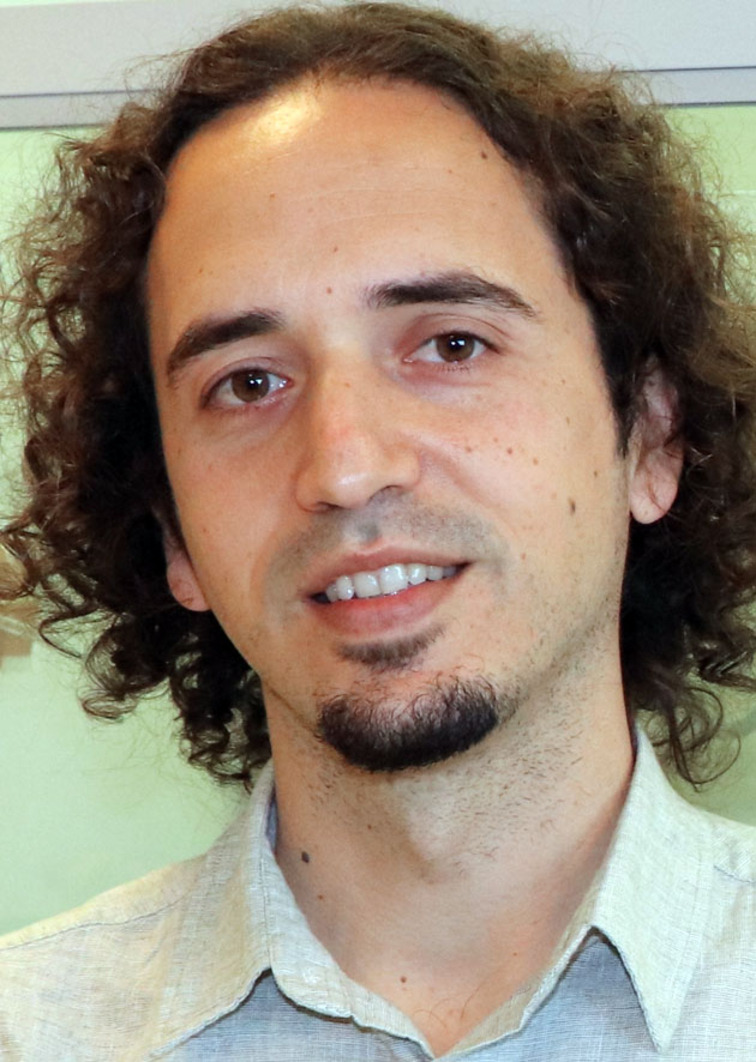# Cooperativity in Transition Metal Tetrylene Complexes

**DOI:** 10.1002/ejic.202100460

**Published:** 2021-08-23

**Authors:** Rosie J. Somerville, Jesús Campos

**Affiliations:** ^1^ Instituto de Investigaciones Químicas (IIQ) Departamento de Química Inorgánica and Centro de Innovación en Química Avanzada (ORFEO-CINQA) Consejo Superior de Investigaciones Científicas (CSIC) and University of Sevilla Avenida Américo Vespucio 49 41092 Sevilla Spain

**Keywords:** Cooperative effects, Group 14 elements, Main group elements, Multi-site activation, Tetrylenes

## Abstract

Cooperative reactivity between transition metals and ligands, or between two metals, has created significant opportunities for the development of new transformations that would be difficult to carry out with a single metal. Here we explore cooperativity between transition metals and divalent heavier group 14 elements (tetrylenes), a less‐explored facet of the field of cooperativity. Tetrylenes combine their strong *σ*‐donor properties with an empty *p*‐orbital that can accept electron density. This ambiphilicity has allowed them to form metal tetrylene and metallotetrylene complexes that place a reactive site adjacent to the metal. We have selected examples to demonstrate what has been achieved so far regarding cooperative reactivity, as this already spans metal‐, tetrylene‐ or multi‐site‐centred bond cleavage, cycloaddition, migration, metathesis, and insertion. We also highlight some challenges that need to be overcome for this cooperativity to make it to catalysis.

## Graphical abstract

Including a divalent heavier group 14 moiety – a tetrylene – within a transition metal complex may at first glance seem rather specialised; however, with the examples collected here, we aim to demonstrate that this has led to a range of fascinating cooperative transformations that have the potential to open up routes to new reactions.

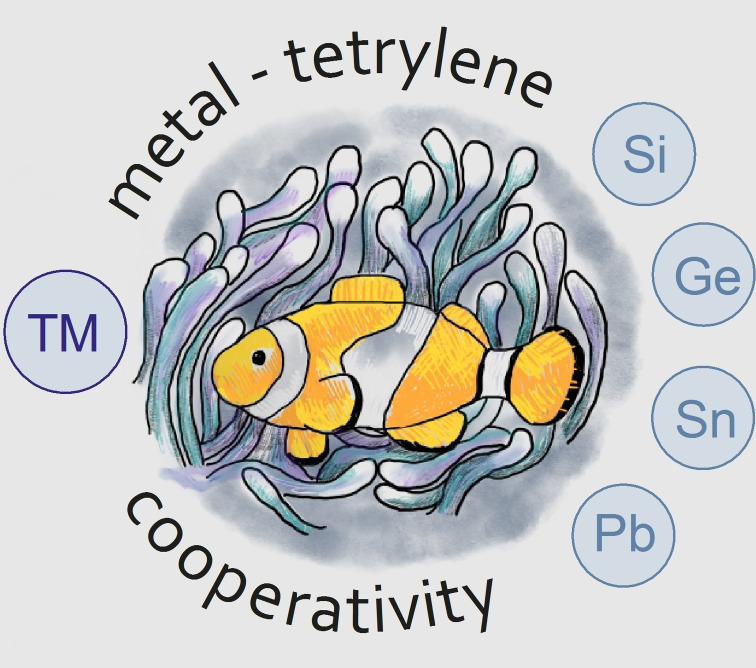

## 1 Introduction

**Figure 1:**
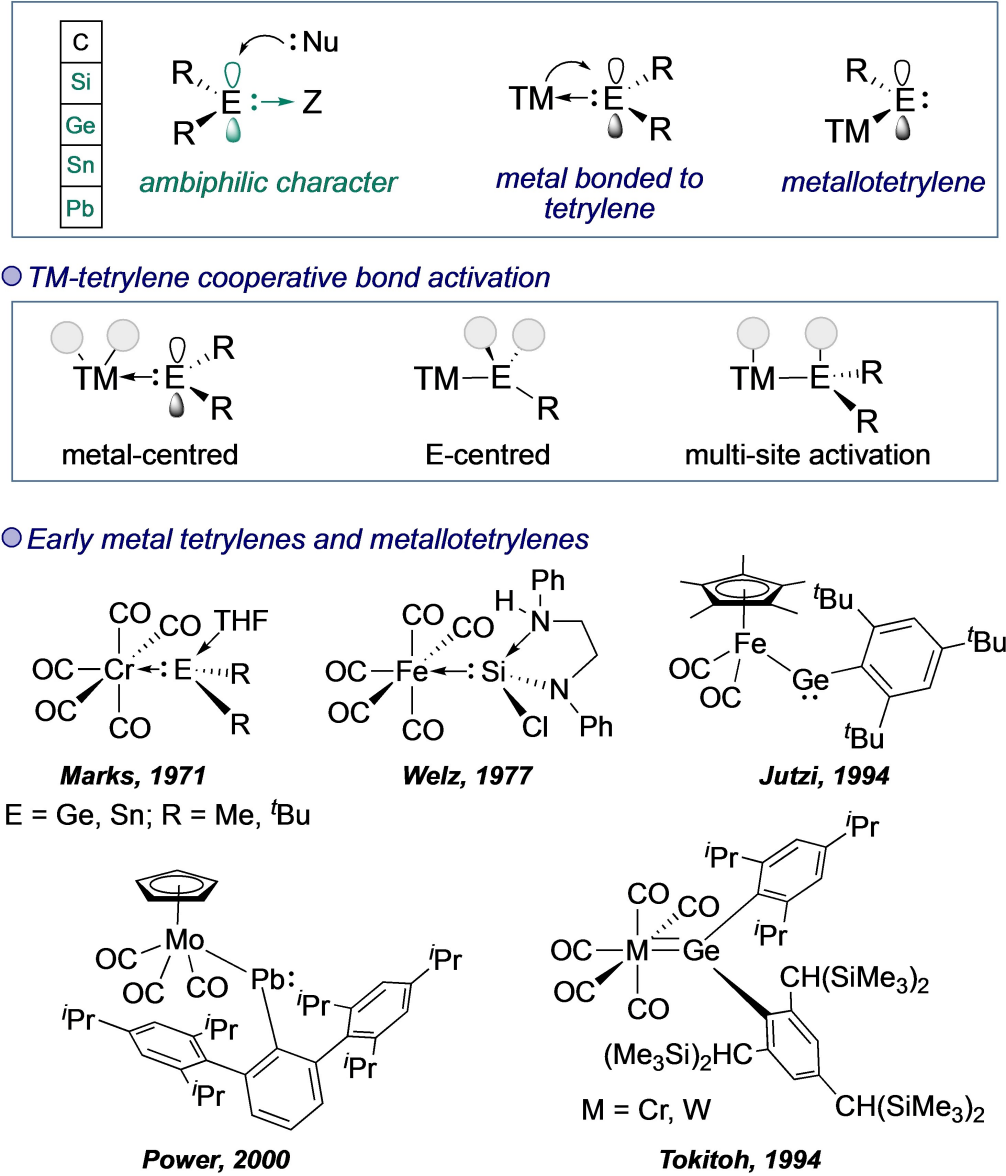
General scheme for metal tetrylenes and metallotetrylenes, early examples, and the cooperative bond activation that could be envisioned between the two centres.

With homogeneous catalysis playing such a pivotal role in organic synthesis, the search for improved catalysts and new reactivity continues at pace. As transition metal complexes make up the vast majority of such catalysts, classical means for their modification through ligand design, the choice of and number of metal centres, and external influences such as light or electric current have received the majority of this research attention.[^1^, ^2^, ^3^, ^4^, ^5^, ^6^] However, studies into ligand designs have also shown that metal‐based reactivity can be influenced by ligand‐based bond‐breaking and bond‐forming, secondary coordination sphere interactions, and ligand‐based redox reactions.[^7^, ^8^, ^9^, ^10^] These are classed as cooperative modes of action. An emerging alternative to these more established classes of non‐innocent ligands is the installation of a single‐site ambiphilic centre that provides reactivity that is complementary to that of the transition metal. In this perspective, we explore the potential of divalent heavier group 14 elements Si, Ge, Sn, and Pb (tetrylenes) as alternative sites for reactivity that occurs between the metal and the ligand (Figure 1).

Although divalent carbon compounds have achieved widespread familiarity as NHC ligands, alkylidene intermediates in metathesis reactions, and as catalysts, heavier tetrylene compounds have found much less uptake.[^11^, ^12^, ^13^] This may be due to a perception that they are rather specialized and limited to a small research community, or a concern that they would be difficult to synthesize or highly reactive. However, although heavier tetrylenes often do require tailored environments such as bulky substituents or stabilization by Lewis bases, it is worth noting that carbenes were also elusive before they were tamed as NHCs.

Interest in heavier tetrylenes lies in their inherent reactivity, which differs from that of divalent carbon compounds.[^12^, ^14^] First, the increased size of heavier group 14 elements and the more diffuse nature of the *p*‐orbitals results in these having a low ability to form hybrid orbitals. This leads to singlet ground states with an unfilled *p*‐orbital that confers electrophilic reactivity and a lone pair of mainly *s*‐character that provides Lewis basicity. This ambiphilicity increases down the group due to the lower *s/p* mixing. Furthermore, combining the *s*‐character HOMO with an additional π‐donor stabilizing group results in tetrylenes being strong σ‐donors that have the potential to be employed as ligands in analogy to NHCs.[^12^, ^15^, ^16^] Importantly, bonds between transition metals and tetrylene partners typically result in a lowering of the HOMO‐LUMO gap of the tetrylene, likely due to the reduced *s‐*character of the HOMO orbital.[^17^, ^18^, ^19^, ^20^] This not only leads to increased reactivity at the tetrylene centre, but to the possibility for cooperativity between tetrylene‐ and metal‐centred reactivity.

The first examples of metal heavier tetrylene coordination (M−ER_2_) to appear in the literature were iron and cobalt‐Sn(II) complexes reported by Hieber, although these were not identified as “stannylenes”, and the platinum dichlorosilylene complex reported by Balk and Schmid.[^21^, ^22^] The beginnings of the field are commonly attributed to Marks’ seminal 1971 report of a chromium dialkylgermylene and two chromium dialkylstannylenes (Figure 1).^[23]^ The ambiphilicity of the low‐valent Ge and Sn centres compared to that of previously reported dialkyl carbene complexes was noted. The first silicon version, a base‐stabilised iron‐dimethylsilylene complex, was reported in 1977, while the first metal‐coordinated plumbylenes – the rarest member of this group – were reported by Lappert.[^24^, ^25^, ^26^] More than a decade later, Jutzi and Leue reported the first fully characterized example of a related and highly interesting set of compounds – the metallotetrylenes. Specifically, this complex was a base‐free metallogermylene with a single bond between Fe and Ge.^[27]^ This type of compound also serves as an entry to tetrylynes,^[28]^ whose reactivity is out of the scope of the present contribution.^[29]^

A common feature in transition metal tetrylene chemistry is the need for kinetic stabilization achieved by steric shielding. Indeed, the use of 2,4,6‐tris[bis(trimethylsilyl)methyl]phenyl groups by Tokitoh,^[30]^ 2,4,6‐tris(*tert*‐butyl)phenyl substituents by Jutzi,^[27]^ and bulky *meta*‐terphenyl groups by Power[^31^, ^32^] has provided a foundation for the development of the field. In the case of terphenyl substituents, our group recently reported a series of structural snapshots that characterize the stabilizing capacity of π‐arene bonding by coordinating a *meta*‐terphenyl germylene to a highly electrophilic gold fragment.^[33]^ Overall, these and other bulky groups have permitted access to otherwise unattainable structures and have made a crucial contribution to the transition from the exploration of new types of metal‐tetrylene coordination and metallotetrylene complexes to investigations into their intriguing reactivity.

Since these works, the chemistry of transition metal complexes of heavier tetrylenes has steadily attracted more attention. Herein, we focus on complexes where cooperativity – meaning *reactivity* at the tetrylene and involvement of the metal centre to form bonds between it and the substrate – is reported. Examples of reactivity that is isolated at the tetrylene are included where it may be relevant to the design of future cooperative transformations. We do not cover the vast amount of work on mainly base‐stabilized tetrylenes acting as donor ligands to transition metals; that is, systems in which the tetrylene behaves as a spectator ligand. We invite the reader to browse a number of excellent reviews to obtain a clearer picture of the coordination chemistry of tetrylenes, their use as strong *σ*‐ donors or as catalysts, and their reactivity in, for example, insertion reactions.[^12^, ^34^, ^35^, ^36^, ^37^, ^38^]

We have organized this minireview by collecting together some representative reports in which elementary reactions take place across the M−E bond or are facilitated by the presence of the two centres, without the aim of presenting a comprehensive vision of all available examples. Most of the work covered herein is based on silylenes and germylenes as these are the most investigated systems. Although several stannylenes are also discussed, to the best of our knowledge there are not yet any examples of the cooperative modes of action described herein for transition metal plumbylenes.[^12^, ^39^] A first section introducing elementary reactions is followed by others based on substrate reactivity. We aim to highlight reports that demonstrate the potential for metal‐tetrylene cooperativity and show that tetrylenes should not be relegated to uses as strong *σ*‐donating ligands, but could also play an active role within a catalyst to facilitate new transformations.

## 2 Elementary reactions and cooperativity

The rich chemistry of transition metals largely relies on their ability to mediate a range of fundamental elementary reactions using their partially‐filled *d*‐orbitals. The ambiphilic character of heavier tetrylenes has led to similar transformations being accomplished that were once believed to be exclusive to transition metals.[^40^, ^41^, ^42^] In transition metal‐tetrylene complexes there is the possibility that elementary reactions take place at either of the two active sites, or at both of them through sequential processes. Bond activation across the TM−E bond is also possible and provides an additional route for cooperativity. Here we briefly describe several selected examples that provide insights into some of the most common elementary reactions. Following this section, we describe the reactivity of different classes of substrates with TM−E bonds, so certain elementary reactions will therefore reappear in later discussions.

### 2.1 Oxidative addition

**Scheme 1:**
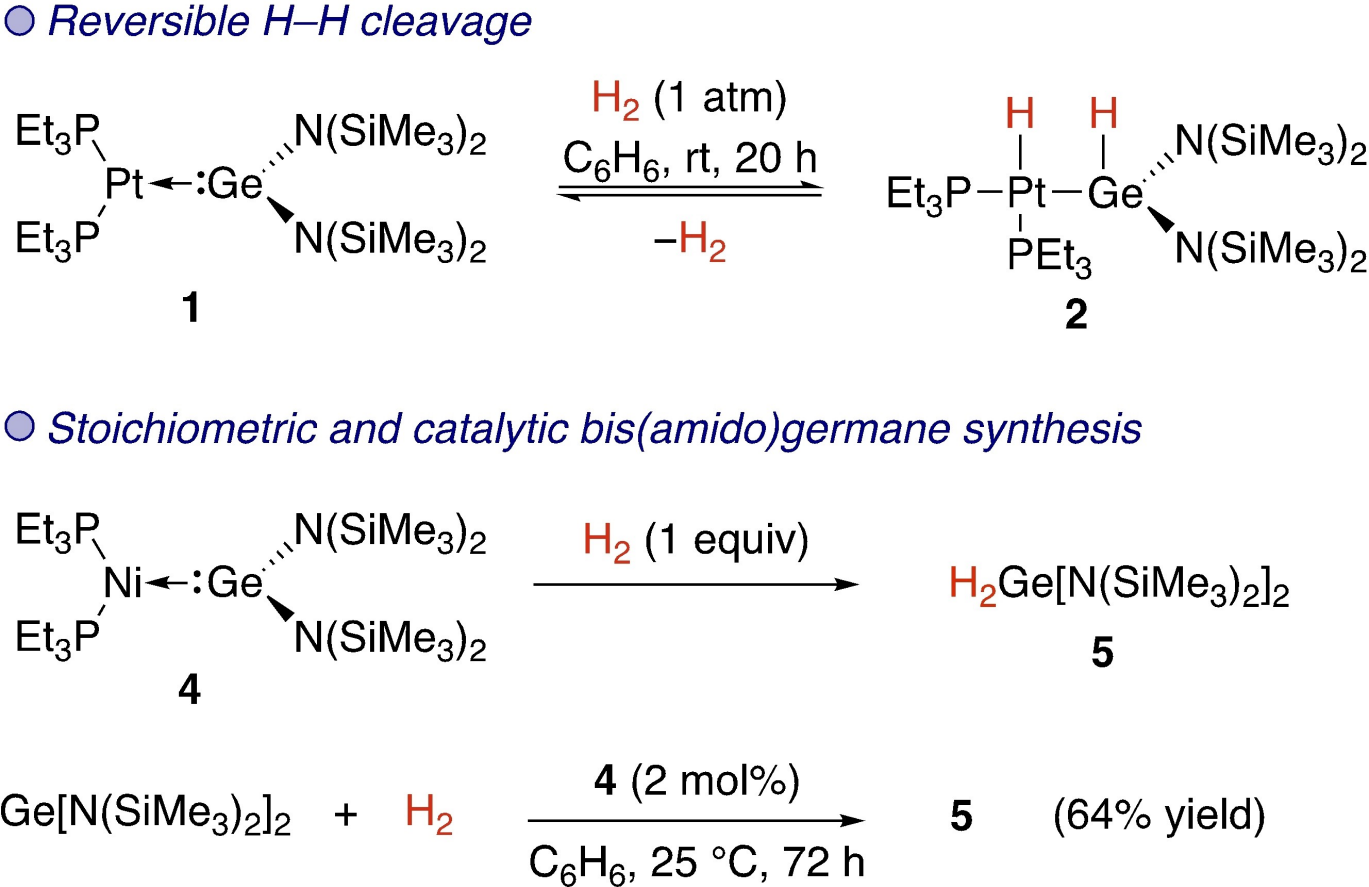
Cleavage of dihydrogen at group 10 metal germylenes.

Foundational work by the Banaszak Holl group explored a series of metal tetrylenes derived from reactions between low‐valent group 10 metal complexes bearing either PEt_3_, PPh_3_ or dppe (1,2‐bis(diphenylphosphino)ethane) and Lappert's germylene (Ge[N(SiMe_3_)_2_]_2_). Cooperative reactivity between the metal and the Ge(II) centre was explored in a series of reports. In their foremost study the Pt(0) germylene complex **1** was reacted with H_2_ (1 atm) at room temperature (Scheme 1).^[43]^ After 20 h, the yellow solution had changed colour to yield the oxidised product **2** that was characterised by X‐ray crystallography. The reaction likely went through a mechanism where oxidative addition at Pt was followed by 1,2‐hydride migration to the germylene ligand. The alternative direct oxidative addition over the Pt−Ge bond was ruled out. Interestingly, the reaction proved to be reversible. Reactions of the palladium analogue (Et_3_P)_2_PdGe[N(SiMe_3_)_2_]_2_ (**3**) with H_2_ were not described, but the nickel analogue **4** was found to be much more reactive, with aminogermane **5** formed directly upon reaction with H_2_ and no complex derived from H−H cleavage observed.^[44]^ In fact, **4** could be used as a catalyst for the synthesis of **5** from H_2_ and the bis(amido)germylene.^[44]^

**Scheme 2:**
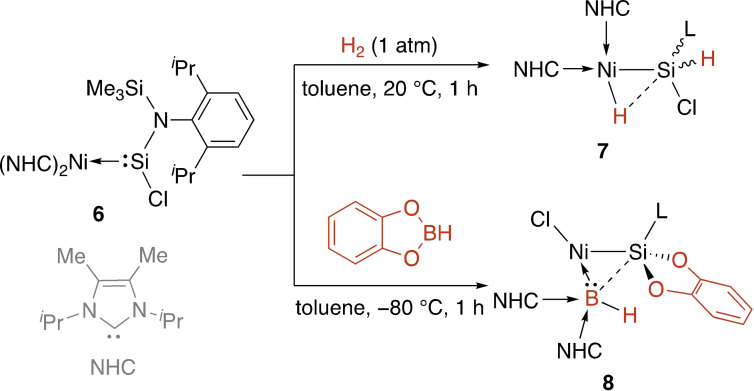
Activation of H−H and B−H bonds at a zero‐valent nickel silylene. L=N(SiMe_3_)(Dipp); Dipp=2,6‐^*i*^Pr_2_C_6_H_3_.

Low‐valent nickel silylenes have been investigated by the Driess group in the context of E−H bond cleavage and the synthesis of heavier analogues of η^2^‐alkene complexes.[^45^, ^46^, ^47^] First, irreversible cleavage of dihydrogen at complex **6** to form nickel silyl complex **7** was observed. The mechanism was proposed to occur in a manner similar to that of Pt complex **1**; that is, nickel‐centred oxidative addition of dihydrogen followed by migration of a H atom to the silicon centre (Scheme 2).^[45]^ In this case, the product contains a Si−H−Ni interaction that supports cooperation between the two sites. In the case of reaction with catechol borane, the remarkable cleavage of the two strong B−O bonds across the Ni−Si bond occurred to generate a monovalent ‘BH’ ligand (**8**). Bond activation of NH_3_, PH_3_, and AsH_3_ was also achieved at **6**, and investigation of further rearrangements provided the first evidence for the tautomerisation between silylene‐silapnictene‐silylpnictinidene compounds, a new class of main group‐transition metal complexes.^[46]^ A somewhat related H−H activation was found at a bis‐silylene Ni(0) complex that was also an efficient hydrogenation catalyst.^[48]^

**Scheme 3:**
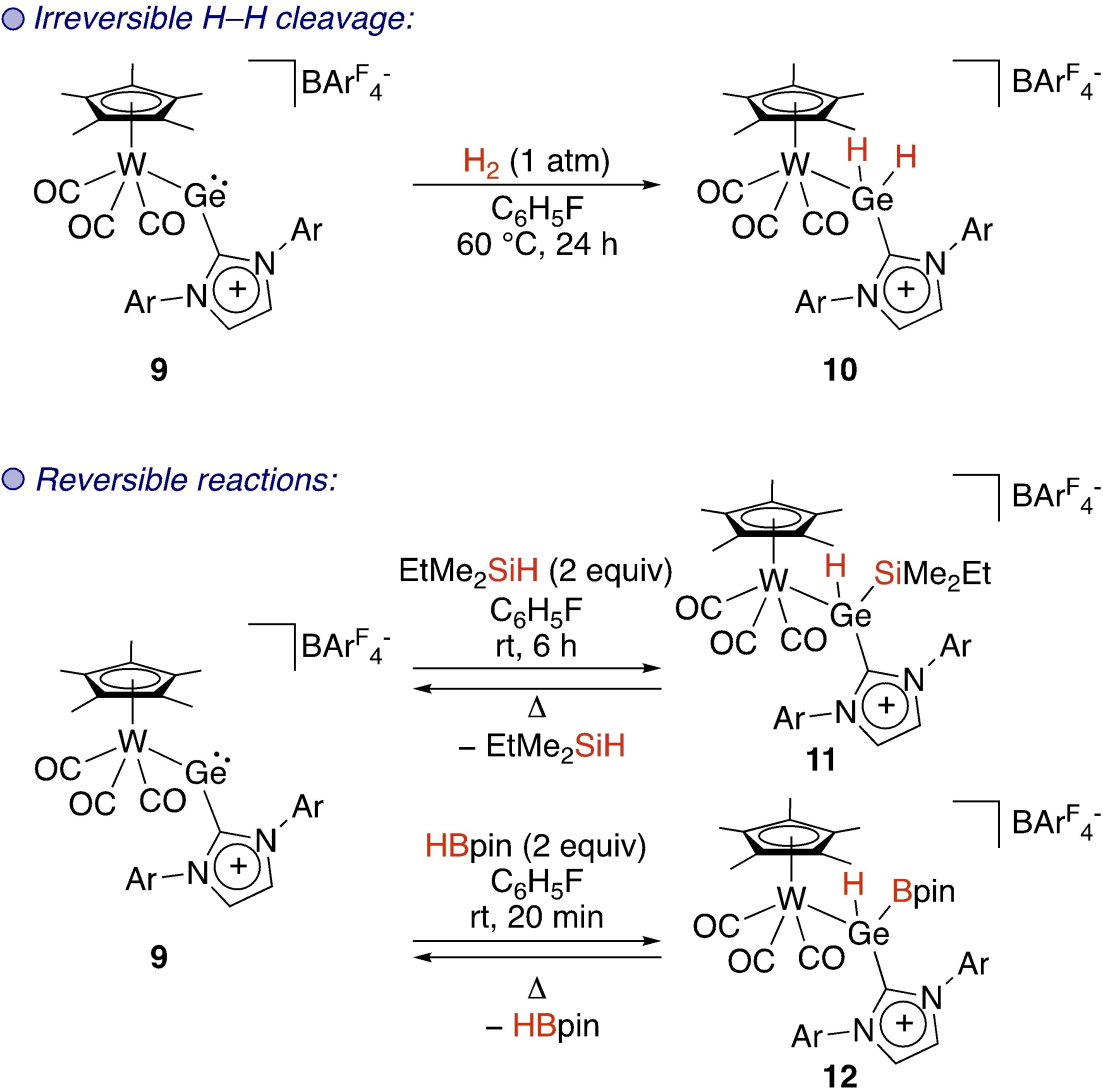
Cleavage of strong bonds at the Ge(II) centre of metallogermylene **9**. Ar=2,6‐^*i*^Pr‐C_6_H_3_, BAr^F^=tetrakis[3,5‐bis(trifluoromethyl)phenyl]borate.

Metallotetrylenes have also proven to be highly reactive towards oxidative bond activation. In these compounds, bond activation may also occur at the tetrel because the presence of the transition metal results in a decreased HOMO‐LUMO gap. Tobita and Hashimoto exploited this effect in cationic Cp*W−Ge complex **9** (Cp*=pentamethylcyclopentadienyl) to cleave E−H bonds in H_2_, silanes, and pinacol borane (HBpin) (Scheme 3).^[18]^ Modest heating was required for reactivity with dihydrogen to form complex **10**, whereas silanes and HBpin reacted at room temperature to form complexes **11** and **12**, respectively. Importantly, the cleavage of Si−H and B−H bonds was found to be reversible. In many cases the Ge(IV) product is a thermodynamic sink, so successful reversibility at this cationic complex containing a M−Ge(II) centre bearing a highly donating NHC sheds light on a potential way of achieving redox (E(II)/E(IV)) catalysis at tetrylenes. Around the same time the Filippou group described the first two‐coordinate metallosilylene.^[19]^ Its ability to oxidatively add a H−E bond in H_2_O, H_2_, HCl, and NH_3_ was demonstrated, albeit in an irreversible manner. The intramolecular insertion of a rhodium metallosilylene into H_2_ and into a sp^2^ C−H bond of a supporting ligand was later reported by Kato and co‐workers.^[49]^

**Scheme 4:**
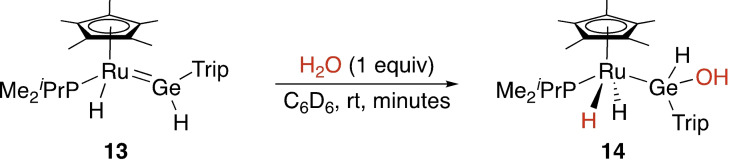
Multi‐site oxidative addition across a metal tetrylene bond. Trip=2,4,6‐^*i*^Pr_3_‐C_6_H_2_.

Beyond single‐site oxidative addition at either the transition metal or the tetrel site, substrate bond cleavage may also occur directly across the M−E bond. A representative example is the activation of water across the Ru=Ge bond in compound **13** to form **14** (Scheme 4).^[50]^ A labelling experiment with D_2_O allowed Tilley and co‐workers to authoritatively support the multi‐site mechanism.

**Scheme 5:**
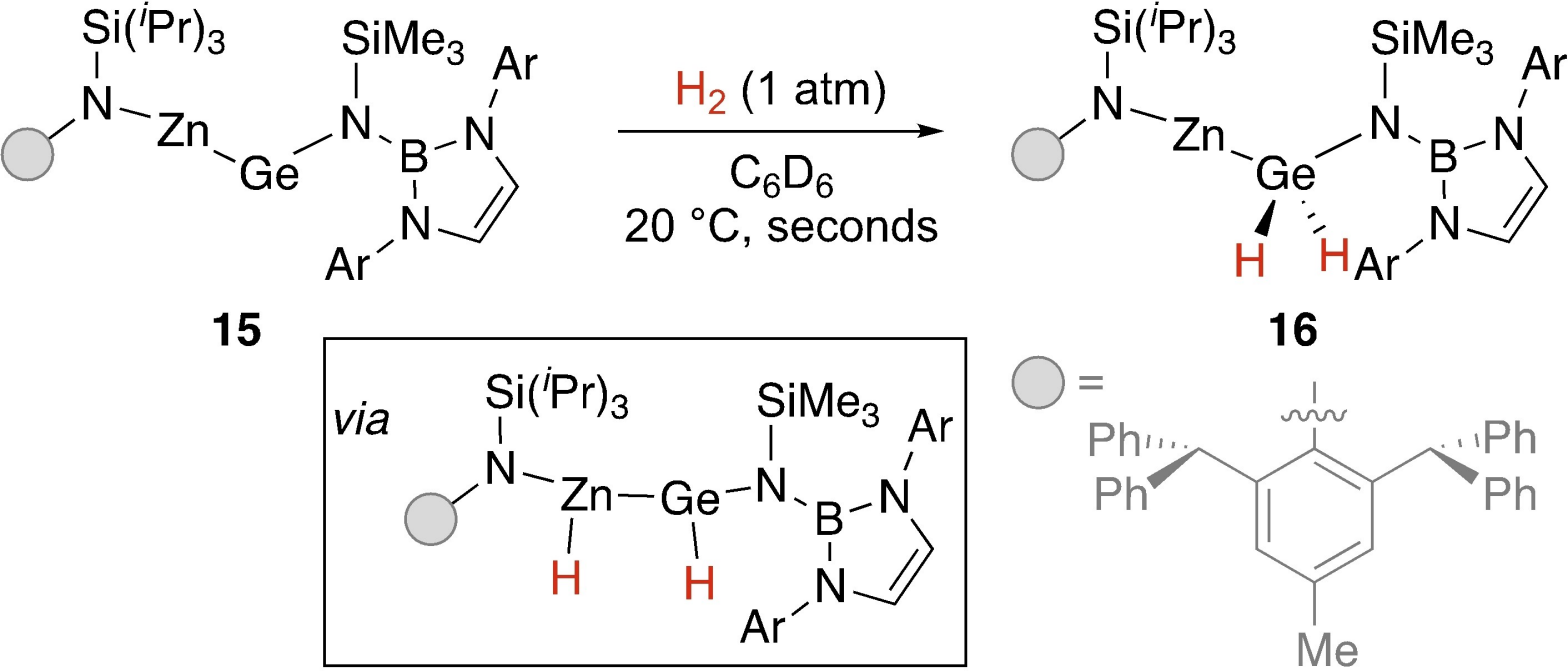
Metallogermylene containing electropositive Zn. Ar=2,6‐^*i*^Pr‐C_6_H_3_.

Jones and co‐workers reported zincagermylene **15** where the electron‐donating metal‐amido group had reduced the HOMO‐LUMO gap at the germanium centre enough for extremely facile activation of dihydrogen.^[51]^ The HOMO and LUMO are significantly germanium‐centred and have a calculated energy difference of 40.8 kcal mol^−1^, much lower than that of **9** (64.1 kcal mol^−1^). Calculations showed that in the first transition state the electron‐poor Zn centre interacts with H_2_ alongside germanium to form the Zn(H)‐Ge(H) intermediate. Hydride migration from Zn to Ge results in the final product (Scheme 5).

**Scheme 6:**
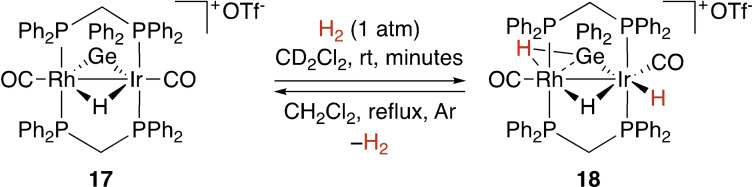
Oxidative addition at a bimetallic Rh−Ir complex.

Oxidative addition reactions may also take place at polynuclear complexes. For example, the Cowie group investigated a rhodium‐iridium platform that can react with primary and secondary silanes or germanes to form tetrylene‐bridged compounds such as **17** (Scheme 6).^[52]^ These can react with hydrogen and HX (X=OH, OMe, H, Cl). Interestingly, the reaction for H−H cleavage shown in Scheme 6 is reversible, while the activation of polar bonds is not. Reaction of **17** with D_2_ demonstrated that exchange of all the hydrides in **18** occurs over 48 h.

### 2.2 Cycloaddition

**Scheme 7:**
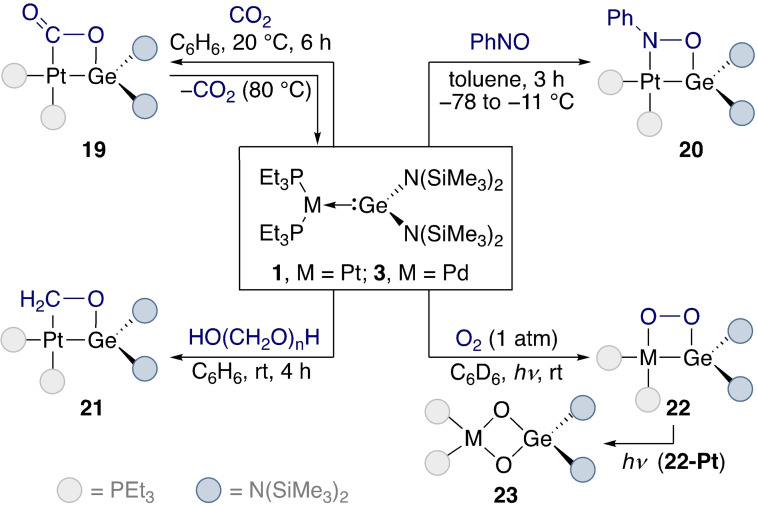
Cycloaddition reactivity of **1**.

The aforementioned systems of type L_2_M−Ge[N(SiMe_3_)_2_]_2_ (M=group 10 metal) developed by Banaszak Holl also exhibit a rich [2+2]‐cycloaddition reactivity which we summarise here as an example of this elementary step. Further examples of cycloaddition reactivity are found in section 3 within the discussion of reactivity with unsaturated substrates. Metal tetrylene complexes **1** and **3** were reacted with CO_2_ (**19**),^[43]^ nitroso compounds (**20**),^[53]^ formaldehyde (**21**),[^54^, ^55^] and O_2_[^54^, ^56^] (**22**) (Scheme 7). In all reactions with **1**, four‐membered metallacycles were formed. Interestingly, when Pt complex **19** was heated at 80 °C, CO_2_ was released.^[43]^ This reactivity contrasts with that of **3** with isoelectronic carbonyl sulfide (COS), where the heteroallene is broken apart and the sulfur atom sequestered in Pd−S−Ge bridges.^[57]^ Going down the group 10 metals, the M−Ge interaction strengthens and the lability of the germylene component decreases. Although Pd complex **3** should therefore be more reactive than **1** towards the above substrates, it was only reactive towards O_2_ and COS.^[56]^ The rearrangement of the side‐bound O_2_ complex **22‐Pt** to bridged species **23‐Pt** required irradiation with UV light, but occurred in the absence of light for **22‐Pd**, similar to a later example containing a Cu(I)–Ge(II) motif.[^54^, ^58^] The nitroso‐derived platinum complexes **20** were thermally stable but light‐sensitive, while reactions with more‐reactive Ni complex **4** resulted in complicated mixtures of compounds.

### 2.3 1,2‐migration

**Scheme 8:**
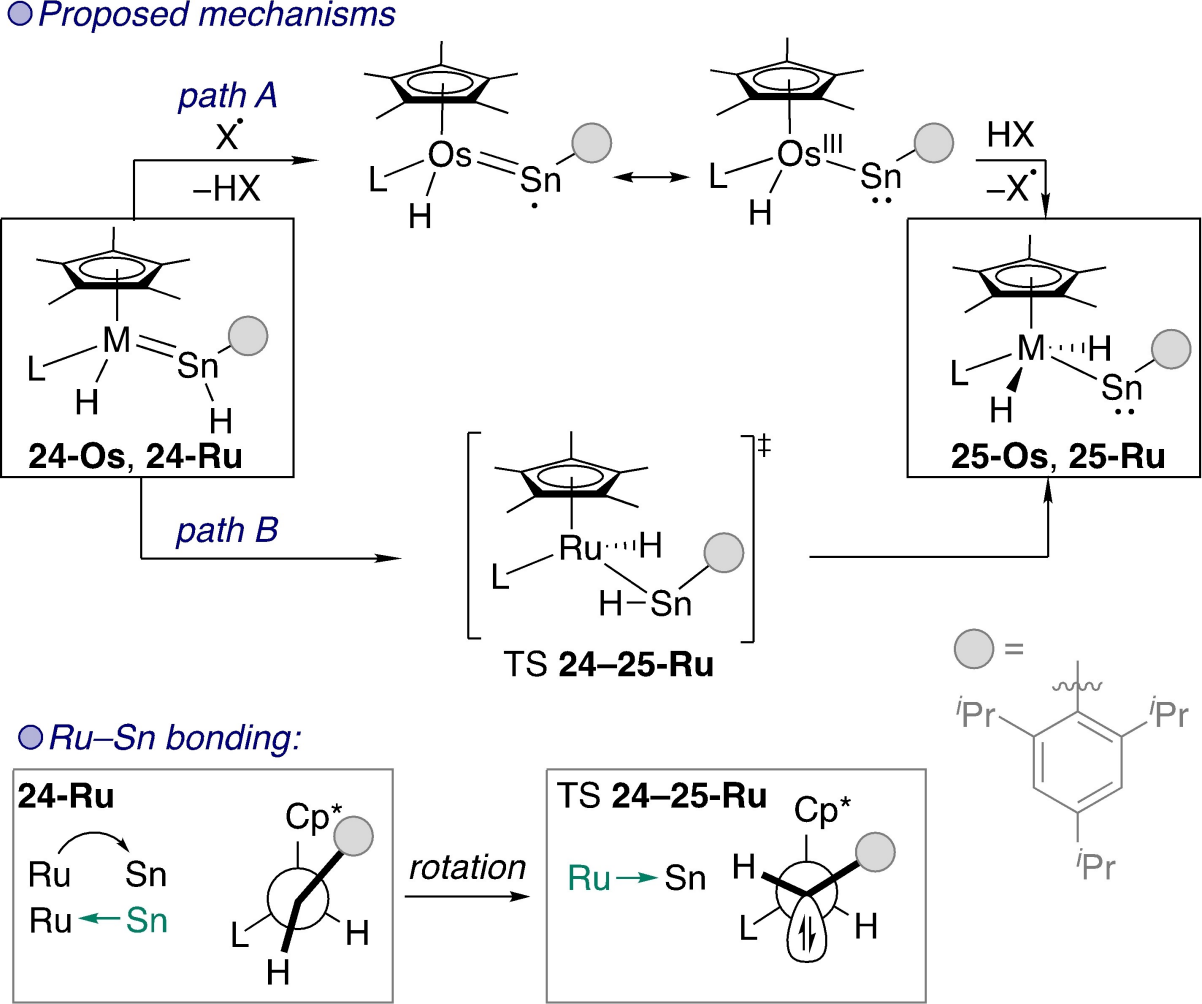
Stannylene to metallostannylene transformations through 1,2‐migration. Os L=P^*i*^Pr_3_; Ru L=1,3‐bis(2,6‐dimethylphenyl)imidazol‐2‐ylidene.

The migration of a hydride between a transition metal and a bound group 14 element, although seemingly a straightforward mechanistic step, involves certain intricacies. The Tilley group studied 1,2‐hydride migration in osmium and ruthenium stannylenes **24‐Os** and **24‐Ru** to form metallostannylenes **25‐Os** and **25‐Ru** (Scheme 8).[^59^, ^60^] There is no empty orbital on the metal centre in either stannylene for conventional 1,2‐migration to occur. For migration in **24**‐**Os** the rate of migration is not affected by excess phosphine, ruling out phosphine dissociation to free up a coordination site.^[59]^ However, radical traps prevent the migration reaction from occurring. A radical mechanism was therefore proposed based on the presence of adventitious radicals (Scheme 8 – path A). This involved alpha‐hydrogen atom abstraction from the Sn centre, then transfer of a H radical back to the Os centre in its tautomeric Os(III) form.

Later, 1,2‐hydrogen atom migration in **24‐Ru** was studied experimentally and computationally (Scheme 8 – path B).^[60]^ In this case, migration was not affected by radical inhibitors and the reaction was found to be first order in the ruthenium stannylene. The mechanism for migration to occur in the absence of an empty orbital on ruthenium was deduced by studying the geometries and bonding interactions in the starting material, product, and TS for the transformation.

In the starting complex **24‐Ru** the Sn(II) site is acting as a two‐electron donor as part of the Ru=Sn double bond. Geometry‐wise, the Sn‐aryl and Ru−Cp* bonds are in an approximately eclipsed conformation. However, in the **24**–**25‐Ru** TS, there is now a *Ru−Sn single bond* and rotation around the Ru−Sn bond has imposed a staggered conformation. The change in bonding interaction means that the donor lone pair of the Sn atom that was taking part in bonding increases its s‐character significantly. Due to the inert pair effect, this orbital does not take part in bonding. It is now the 5*p*‐orbital on Sn that is *accepting* electrons from an orbital on ruthenium as part of the Ru−Sn bond. The change in bonding from a double bond to a donor‐acceptor interaction opens the pathway for hydride transfer via an agostic interaction between the polarised Sn−H bond and the Ru centre.

The Os=Sn bond in **24‐Os** has a greater covalent character, preventing the change in the metal‐tetrylene bonding interaction that facilitates migration in **24‐Ru** and thus proceeds through a different – radical – mechanism. Tobita developed a related iron complex that displays a germyl‐germylene equilibrium. The propensity of the complex towards 1,2‐migration can be controlled by the addition of weak nucleophiles such as nitriles and pyridine.^[61]^

**Scheme 9:**
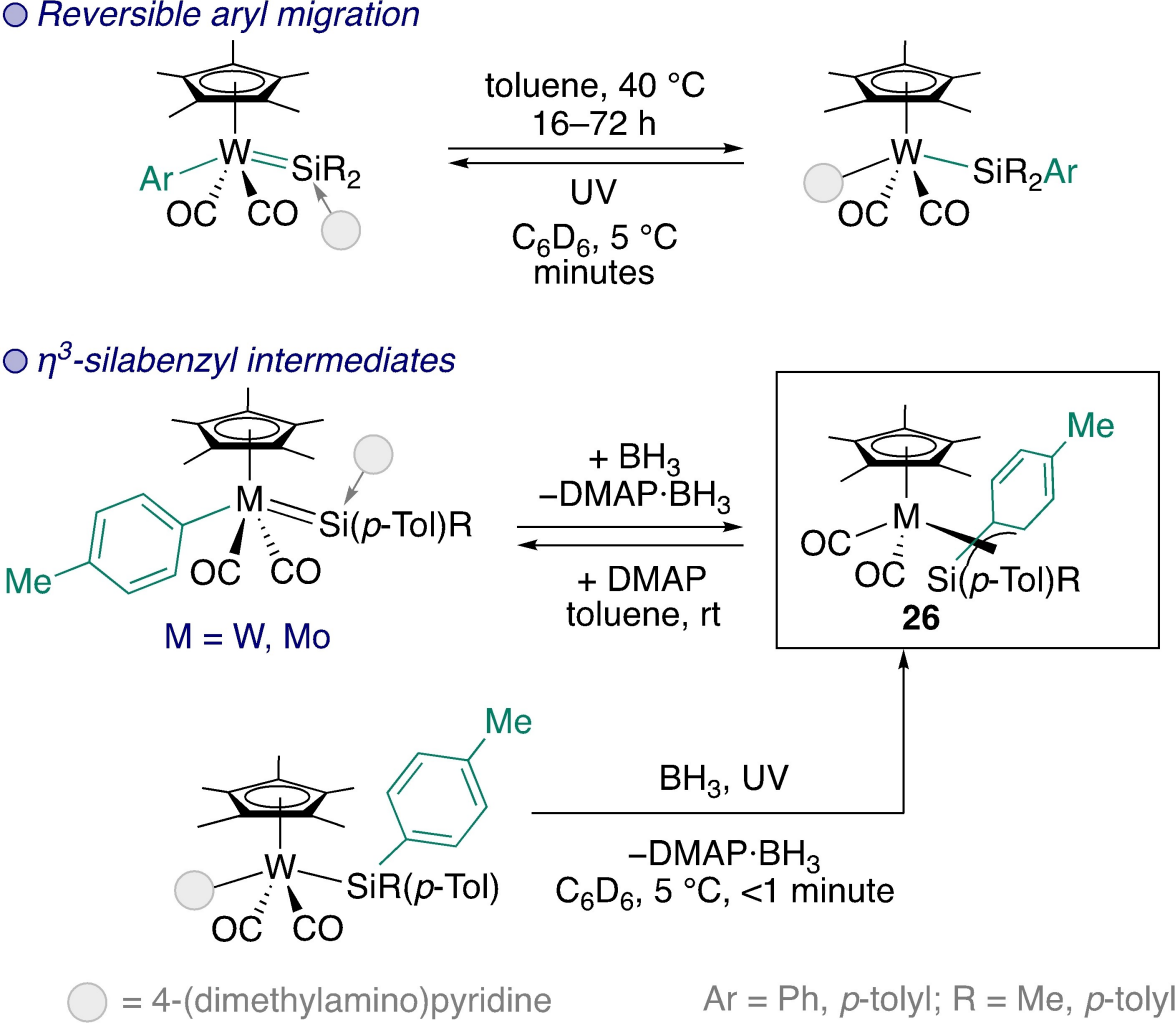
Reversible 1,2‐aryl migration and synthesis of silabenzyl intermediates.

As a first step to demonstrating migration with larger groups, the Tobita group investigated 1,2‐methyl and 1,2‐aryl migration from W to Si in a range of silylenes.[^62^, ^63^, ^64^, ^65^] Work on reversible aryl migrations, which is summarised in Scheme 9, resulted in the isolation of the aryl migration intermediate – a silicon analogue of an η^3^‐benzyl complex (**26**) – for both W and Mo.[^64^, ^65^]

### 2.4 Metathesis

**Scheme 10:**
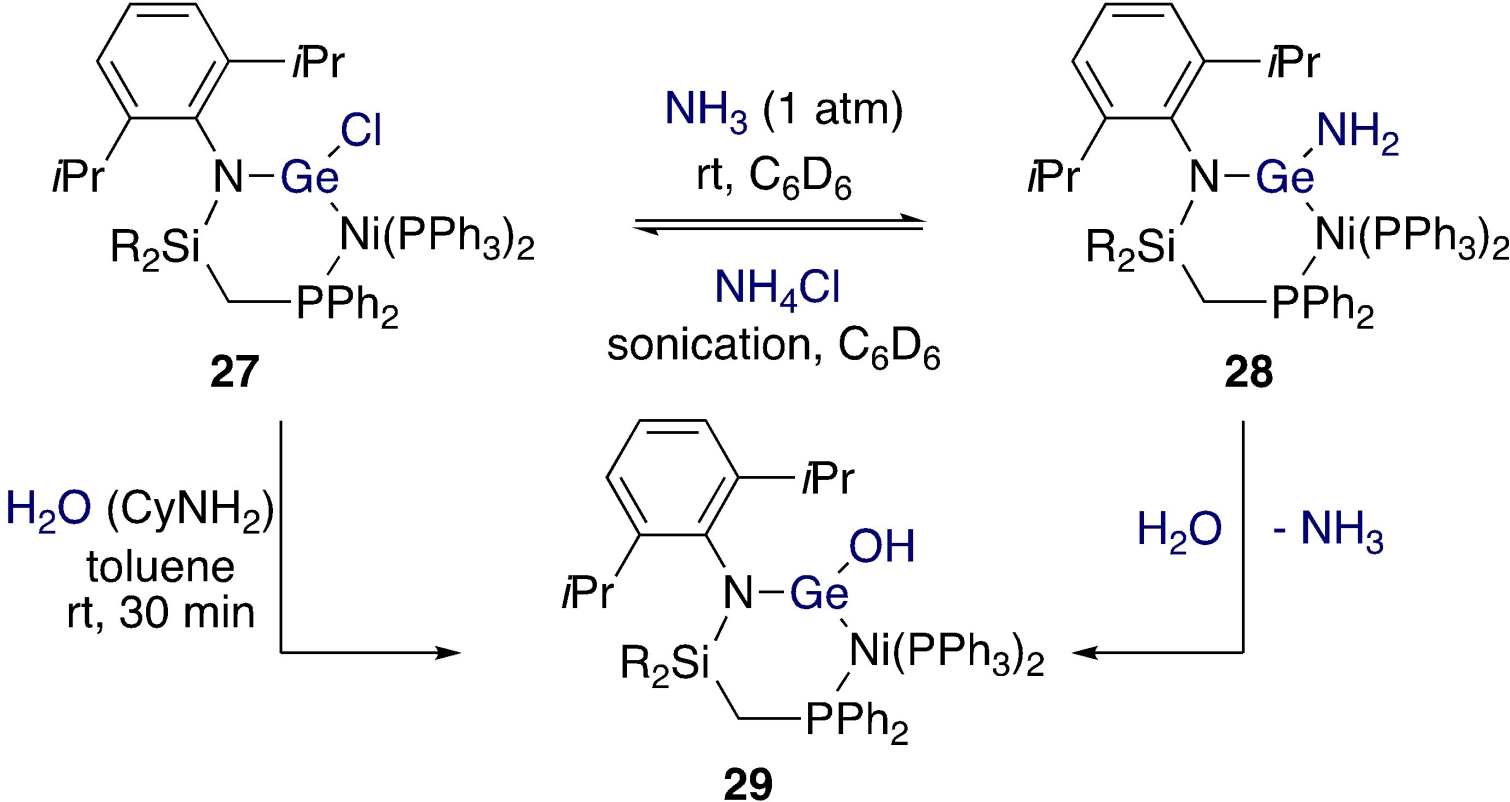
Reversible metathesis at Ge(II) without direct involvement of the transition metal R=Ph, ^*i*^Pr.

Hadlington and co‐workers recently employed a Ge(II)−Cl moiety as a site for reversible reactivity in a transition metal complex (Scheme 10).^[66]^ Although reactivity only occurs at the germanium centre, it is important to include this example here because it proposes a way of maintaining the Lewis acidity of the germylene component rather than, as in the majority of the examples herein, forming Ge(IV) and losing its ambiphilicity. Nickel complexes were synthesized where a chelating phosphagermylene ligand brought the Ge−Cl bond and the nickel centre into close proximity. Nickel halide precursors resulted in germyl complexes, while reduction of these or the direct use of a Ni(0) precursor resulted a Ni(0) complex containing trigonal planar Ge(II) (**27**). A DFT study showed the presence of the desired vacant *p*‐orbital on Ge and indicated that the Ni−Ge bond is polarized towards Ge and has some π bonding character. The Ge−Cl bond reacts with NH_3_ to form Ge−NH_2_ complex **28** and HCl. Oxidative addition did not occur at the Ni centre in **27** because it is coordinatively saturated and the Ge(II)−Cl moiety is coordinated to Ni. Calculations suggested a mechanism that requires two additional NH_3_ molecules to form a hydrogen bonding network between the N−H and Ge−Cl bonds. The reaction is reversible, as supported by calculations showing an energy difference between the Ge−NH_2_ and Ge−Cl species of only −0.9 kcal mol^−1^. Further reactivity of **28** towards water gave Ge−OH complex **29**, again without reactivity at Ni. This complex was also accessed by reacting **27** with an undried amine base.

## 3 Reactivity with unsaturated substrates

Unsaturated molecules such as alkenes and alkynes, carbonyl compounds, heteroallenes, and nitriles are key building blocks in catalytic (and stoichiometric) reactions carried out at transition metals.[^67^, ^68^] This makes them excellent molecules with which to investigate bond‐forming and bond‐breaking reactivity. As is described below, a variety of reactivity is possible with these substrates, having important catalytic implications.

### 3.1 Alkenes and alkynes

**Scheme 11:**
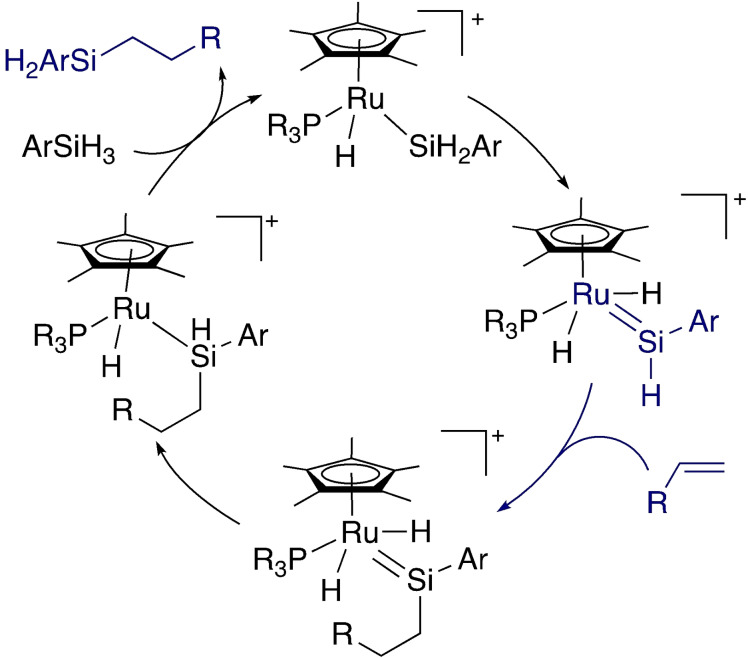
Glaser‐Tilley mechanism for alkene hydrosilylation.

The Tilley group has played a key role in developing the chemistry of metal tetrylenes with unsaturated systems.^[69]^ This has been built up from an extensive understanding of late transition metal silylenes. Reactivity with unsaturated systems has often been framed in the context of hydrosilylation reactions. The Tilley group developed catalysts that offered an alternative silylene‐based hydrosilylation mechanism to the previously‐known platinum catalysts. Specifically, the Glaser‐Tilley mechanism involves selective insertion of the alkene into the Si−H bond of the silylene, rather than coordination of the alkene to the metal followed by migration (Scheme 11).^[70]^ The silylene is regenerated by α‐hydrogen migration, a key elementary step already introduced above. Not only did this work provide an understanding of the mechanism of the catalytic reaction by developing metal silylene complexes, it provided an understanding of fundamental features of reactivity that might be applicable to cooperative systems. Hydrosilylation reactions involving silylenes of a range of group 8 and 9 metals have been investigated through the synthesis of complexes, mechanistic studies, stoichiometric reactions, and computational investigations.[^70^, ^71^, ^72^, ^73^, ^74^, ^75^]

**Scheme 12:**
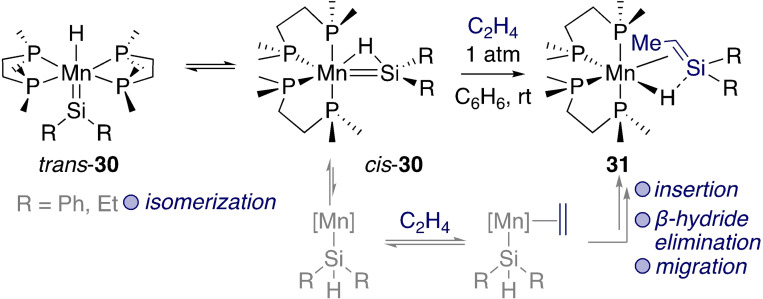
Ethylene insertion at Mn silylene hydride complexes.

The Emslie group has also provided valuable fundamental knowledge on the reactivity of silylenes with alkenes.[^76^, ^77^] For instance, the reaction of manganese silylene hydride complex *cis*‐**30** with ethylene formed a complex bearing a coordinated silene ligand (**31**) (Scheme 12).^[76]^ Direct insertion of ethylene into a Mn−H bond was ruled out, and a [2+2] cycloaddition mechanism was not supported by either calculations or stoichiometric experiments. An alternative mechanism was proposed where a key Mn‐silyl complex derived from *cis‐* **30** opened a coordination site for ethylene binding and a series of 1,2‐insertion, elimination, and migration steps.

**Scheme 13:**
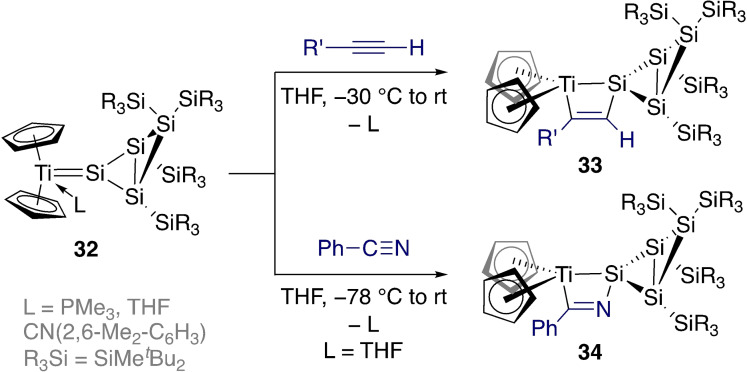
Schrock‐type titanium silylenes in [2+2] cycloadditions.

Lee, Sekiguchi, and colleagues have drawn parallels between Schrock's alkylidene metathesis catalysts and the possibility for the development of silicon‐based metathesis systems (Scheme 13).^[78]^ In this case, a [2+2] cycloaddition between titanium silylene **32** and terminal alkynes formed silatitanacyclobutenes **33** as single regioisomers. The mechanism was investigated computationally and was found to favour pre‐coordination of the alkyne to Ti prior to the cycloaddition. Unfortunately, no cycloreversion products were detected, and further studies are required to understand the limits to Si‐based metathesis at Schrock‐type tetrylenes. Following up on this work, Sekiguchi and coworkers also reacted **32** with benzonitrile to form metalacyclic complex **34**.^[79]^

**Scheme 14:**
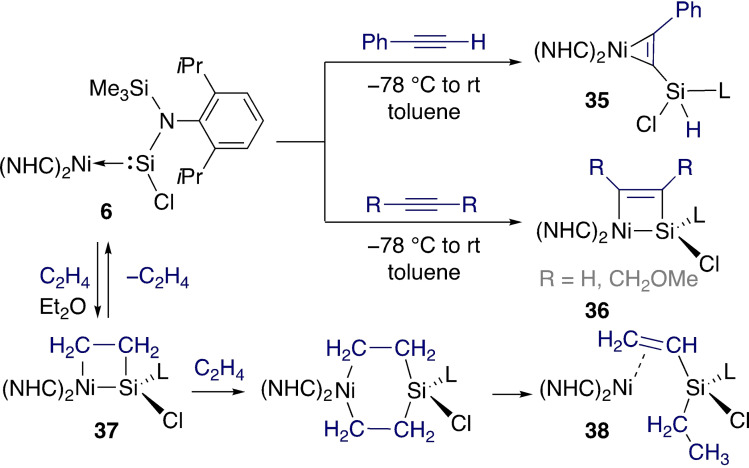
Reactions of unsaturated compounds with a low‐valent nickel silylene.

Similar [2+2] cycloaddition reactions between nickel silylene complex **6** and a range of unsaturated compounds were studied (Scheme 14).^[80]^ In the case of phenylacetylene its acidic C−H bond led to C−H activation across the Ni=Si bond (**35**), while internal alkynes led to the corresponding [2+2] cycloaddition product (**36**).

Interestingly, the reaction with ethylene was reversible. After 24 h at room temperature, a solution of the resulting nickelasilacycle **37** converted back to a mixture of **6** and a new Ni(0) compound **38**. This resulted from the reaction of the liberated ethylene with **37** to form a six‐membered ring, followed by β‐hydride elimination and reductive elimination. Related transformations were carried out with polar unsaturated substrates (e. g. imines, aldehydes); in these cases the proposed mechanism is a concerted [2+2] cycloaddition. In contrast, ethylene (and alkynes) first react at nickel to form a metallacyclopropane, followed by ring expansion towards the nickelasilacyclobutane.

### 3.2 Carbonyl compounds and nitriles

**Scheme 15:**
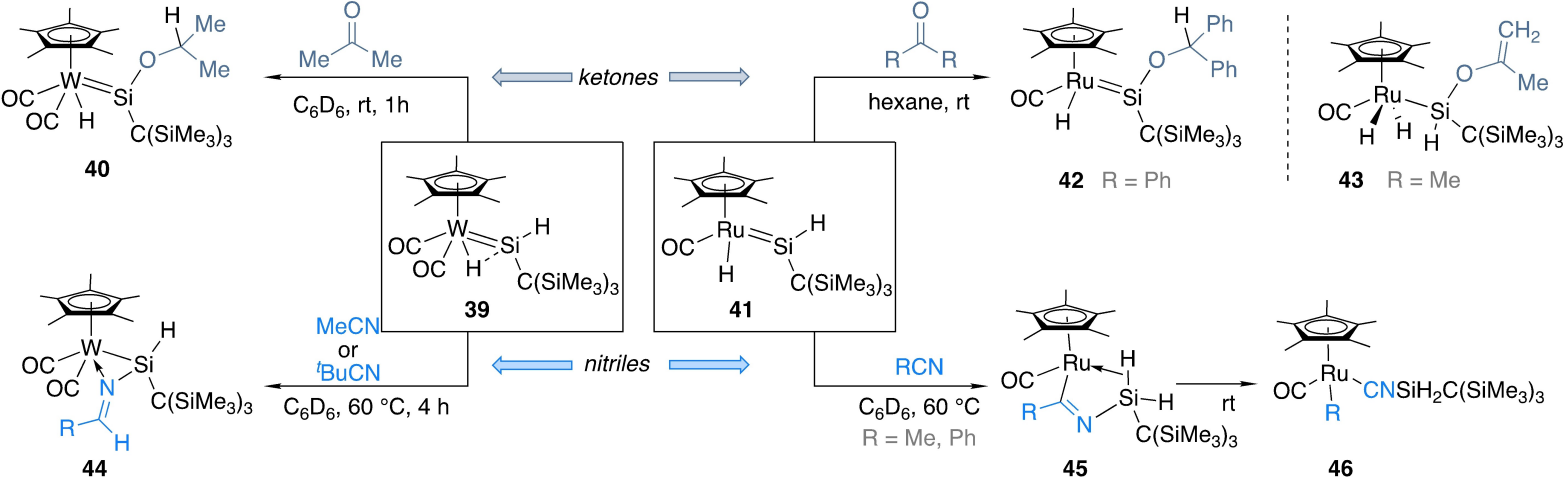
Reactivity of metal hydrido(hydrosilylenes) towards carbonyl compounds and nitriles.

Alongside Tilley's ruthenium silylene complexes, Tobita and colleagues developed a number of isolable hydrido(hydrosilylene) and hydrido(hydrogermylene) complexes as part of their work on tetrylene complexes.[^75^, ^81^, ^82^, ^83^] For example, tungsten complex **39** was reacted with acetone and the product of the formal hydrosilylation of acetone formed as the major species (**40**, Scheme 15).^[83]^ This initial hint of reactivity was later studied in more detail to gain mechanistic knowledge.[^84^, ^85^] These studies suggested that compound **40** was formed after coordination of acetone to the electrophilic silylene followed by hydride migration from tungsten to the carbonyl carbon and subsequent isomerization. The formation of other minor or transient species based on W/Si cooperation was also investigated.

Reactivity towards ketones was investigated for the related ruthenium complex **41**.^[82]^ In this case divergent reactivity was found depending on the substrate. Although ketones without α‐hydrogen atoms led to the same hydrosilylation products (**42**) as the major species, those containing α‐hydrogen atoms yielded α‐hydrogen abstraction species **43**. In both cases cooperation between Ru and Si centres was crucial. Hydrido(hydrosilylene) complexes **39** and **41** were also reacted with nitriles to demonstrate stoichiometric hydrosilylation of substrates that are not particularly activated towards this reaction.[^86^, ^87^] Again, different products formed based on the metal. With tungsten complex **39**, hydrosilylation occurred to form **44**, a complex containing a three‐membered W−Si−N ring, with the W−Si bond lengthened from that in **39** and the Si−N bond being close to a single bond. The reaction was proposed to occur via coordination of the nitrile to the electrophilic Si atom followed by hydride migration from tungsten to the nitrile carbon and subsequent coordination of the lone pair of the nitrogen atom to the coordinatively unsaturated tungsten. In the case of **41**, nitriles did not undergo hydrosilylation. Instead, iminoacyl compounds **45** were formed. As observed for other related systems, these readily converted into silylisocyanides **46** after C−C cleavage.[^88^, ^89^]

**Scheme 16:**
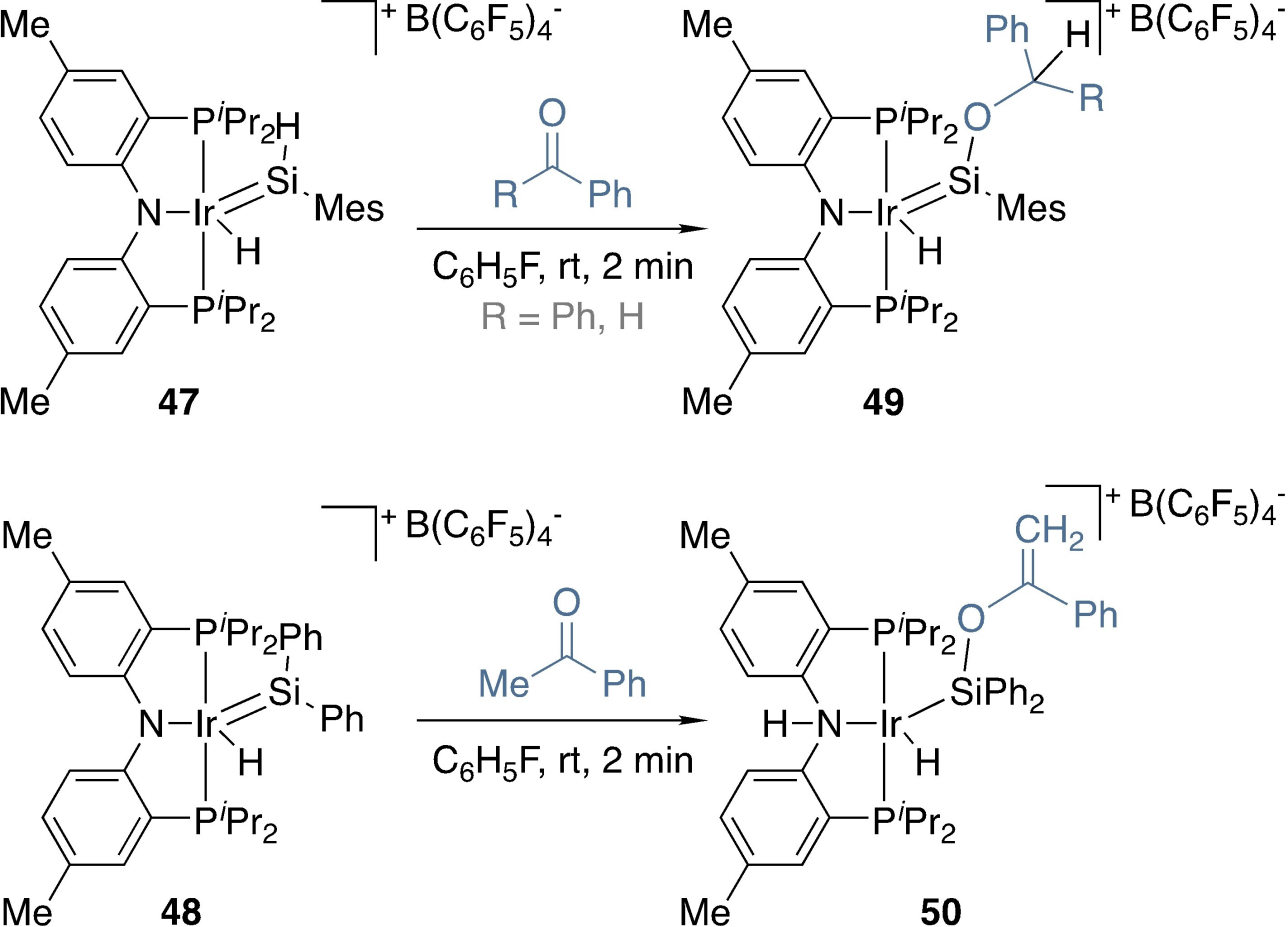
Reactivity of pincer silylene complexes towards simple ketones.

Highly reactive metal tetrylene complexes are not limited to piano stool complexes. For example, the Tilley group synthesized related iridium complexes bearing a PNP pincer ligand (**47** and **48**, Scheme 16).[^90^, ^91^, ^92^] These were reacted with carbonyl compounds, alcohols, amines, and with alkenes in the context of their ability to catalyse hydrosilylation reactions..[^90^, ^91^] As for Scheme 15, carbonyl compounds underwent diverging transformations depending on the absence or presence of α‐hydrogen atoms to form **49** and **50**, respectively.^[92]^ Cooperative reactivity extended to the PNP ligand, which ended up protonated for acetophenone (**50**), dimethylformamide, and after aminolysis or alcoholysis of **48**.

### 3.3 Reactions with Heteroallenes

**Scheme 17:**
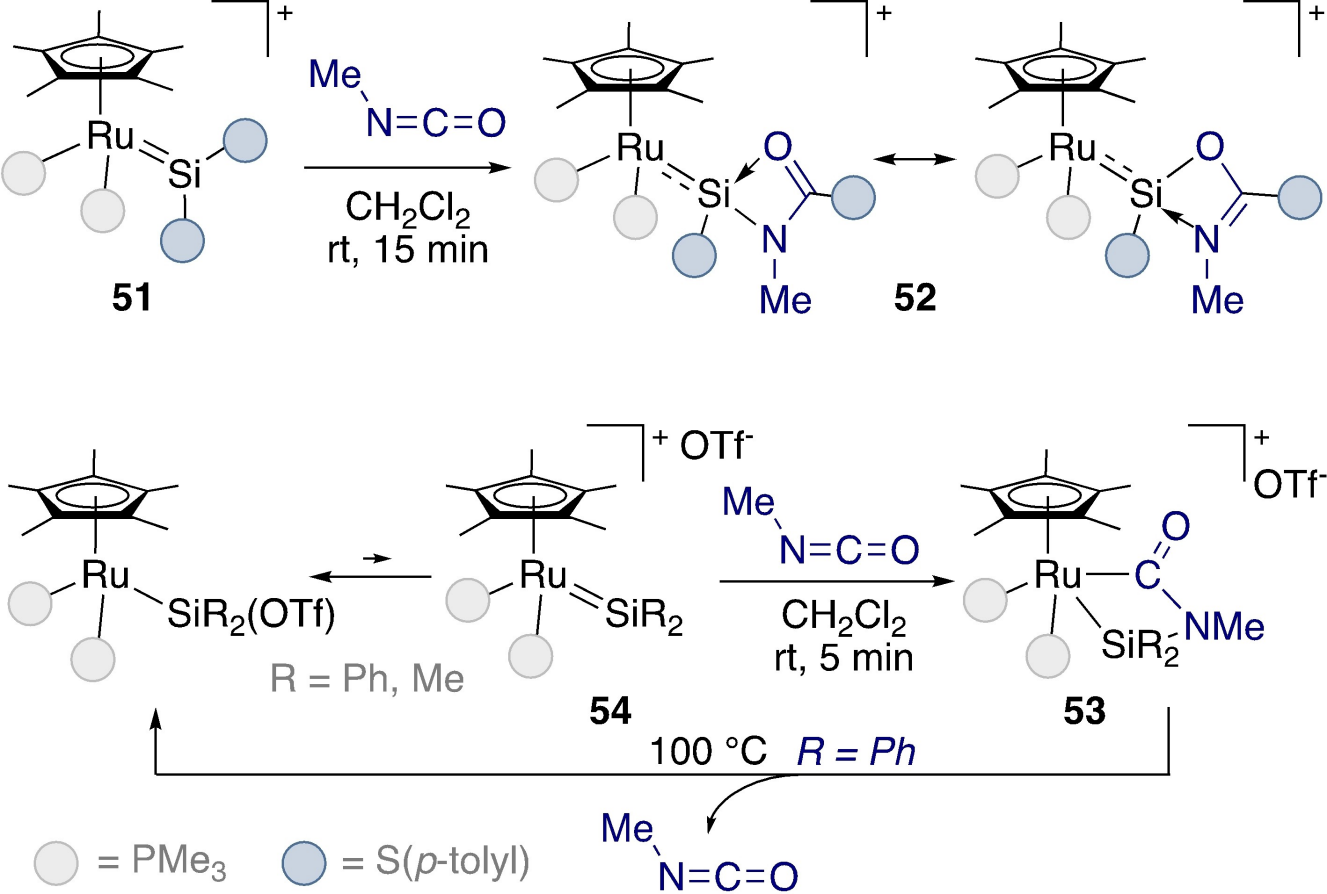
Cycloaddition reactions at ruthenium silylenes.

The Tilley group demonstrated the first cycloaddition of isocyanates to isolated ruthenium silylene complexes (Scheme 17 – top).^[93]^ The proposed mechanism was termed a stepwise cycloaddition, with coordination of the isocyanate to the electrophilic silicon centre of **51** bringing the C=N bond into close proximity to the electron‐rich ruthenium centre on the way to **52**. The reaction was more rapid for electron‐rich isocyanates, as this promoted coordination of the isocyanate to the silylene. Interestingly, complex **53** derived from cationic silylene **54** released methylisocyanate upon heating at 100 °C.

**Scheme 18:**
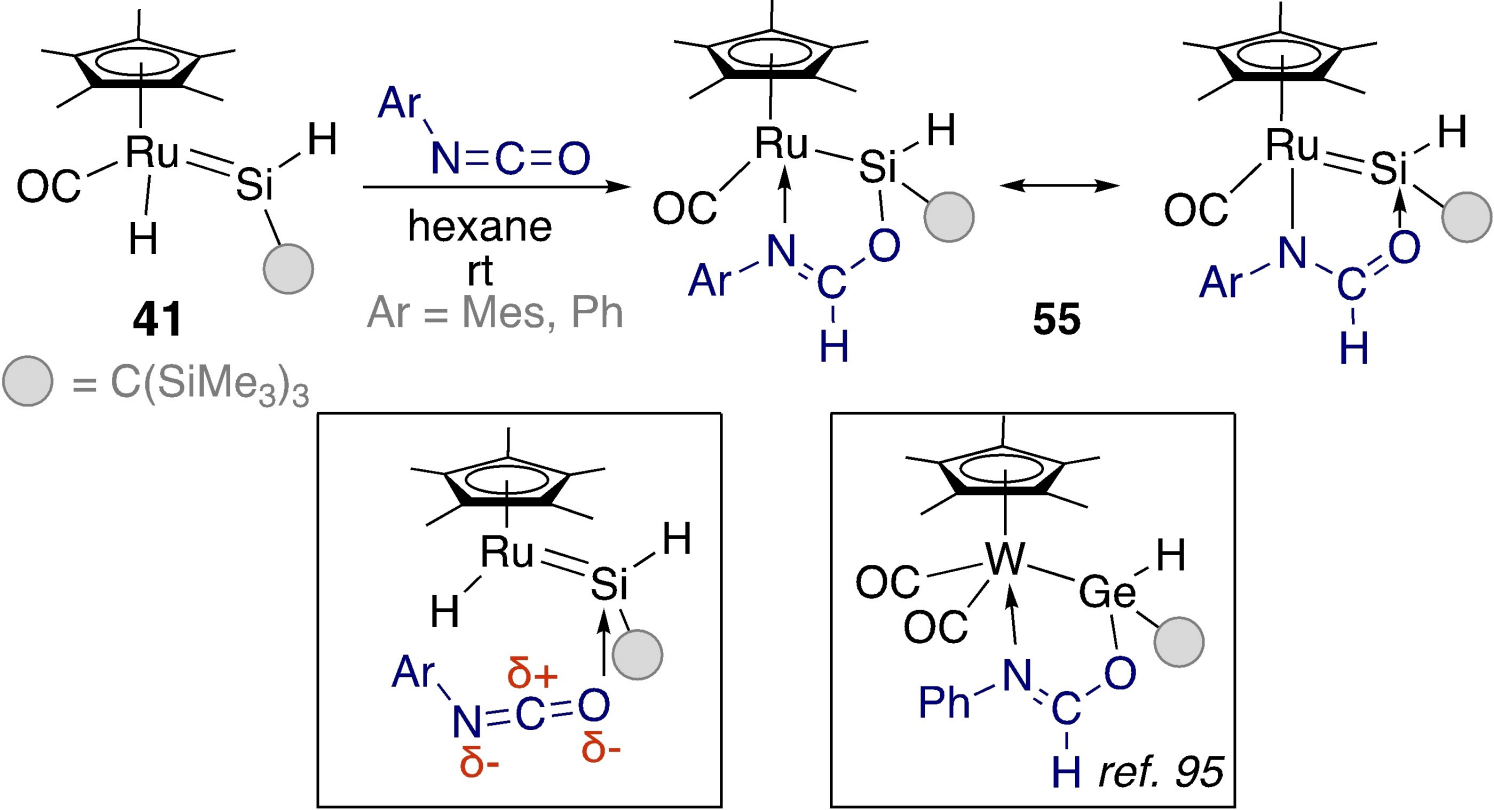
Isocyanate reactivity at ruthenium hydrido silylenes.

**Scheme 19:**
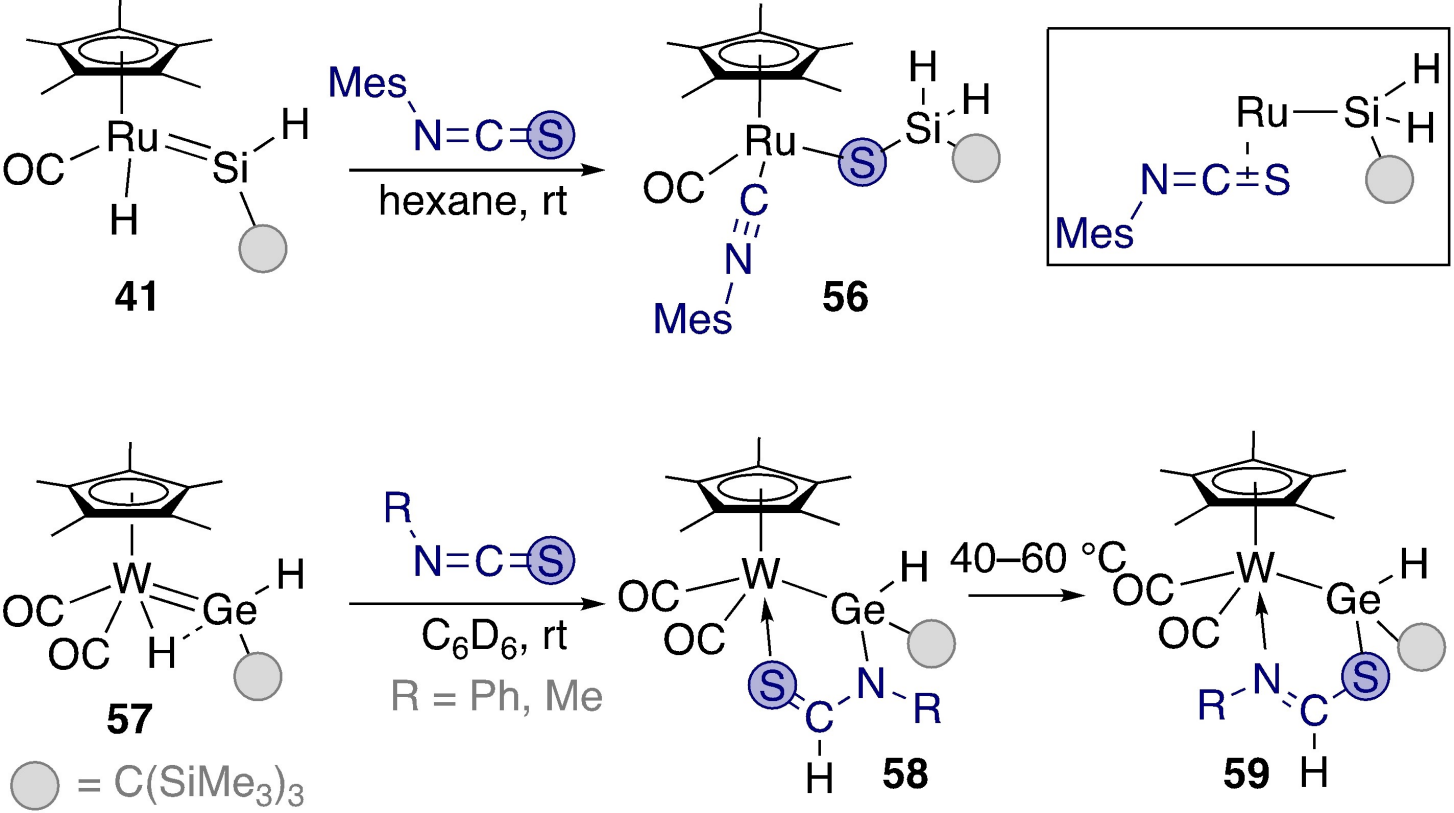
Divergent reactivity of Ru and W tetrylenes towards isothiocyanate.

The Tobita group also subjected ruthenium hydrido silylene **41** to reactions with isocyanates and isothiocyanates (Scheme 18 and Scheme 19, respectively).^[94]^ Again, the initial step for this reaction involved coordination of the isocyanate to the silicon atom. However, the presence of the Ru−H bond – in comparison to Tilley's bis(phosphine) complex – opened the possibility for hydride migration to the electrophilic carbon atom of the coordinated isocyanate. Rotation and hydride migration forms **55**, with resonance contributions of a silylene and a Ru‐silyl that is the product of formal hydrosilylation of the C=O bond. Comparable hydrogermylation reactivity was observed for the germylene analogue of tungsten complex **39** with phenyl isocyanate.^[95]^

Reactivity of **41** with isothiocyanates resulted in C=S bond cleavage rather than hydrosilylation (**56**, Scheme 19 – top). This reactivity was also reported for an analogous iron germylene.^[61]^ The mechanism of the transformation was studied computationally by Xie and Lin.^[96]^ First, the sulfur atom binds less strongly to the silicon centre than oxygen. This disfavours a route similar to that of the isocyanates described above where coordination of the substrate to the silicon centre through sulfur would facilitate hydride migration from ruthenium to the carbon atom of the Ar−N=C=S molecule. Secondly, the C=S bond is a stronger π acceptor than the C=N bond, resulting in the isothiocyanate's increased affinity for the metal centre compared to the isocyanate. Coordination of the isothiocyanate to ruthenium occurs prior to hydride migration from ruthenium to the electrophilic silylene. This is followed by a [2+2] cycloaddition that cleaves the C−S bond.

Cleavage of the C=S bond was not observed for reactions of tungsten germylene complex **57** with phenyl and methyl isothiocyanate.^[95]^ Instead, hydrogermylation of the substrate was observed, in analogy to the reactivity of ruthenium silylene **41** with isocyanates previously described in Scheme 18. Although there may be many factors influencing the divergent reactivity, favourable coordination of the isothiocyanate via the nitrogen atom to the germylene prior to hydride migration may be an important contribution in the latter case. Besides, the W=Ge bond in **57** is also slightly more polarised than the Ru=Si bond of **41**. The electronegativity difference between S and O also meant that the W←S kinetic product **58** rearranged to the N‐coordinated complex **59**.

Overall, the reactions described above require the cooperative participation of both the transition metal and the tetrylene.

**Scheme 20:**
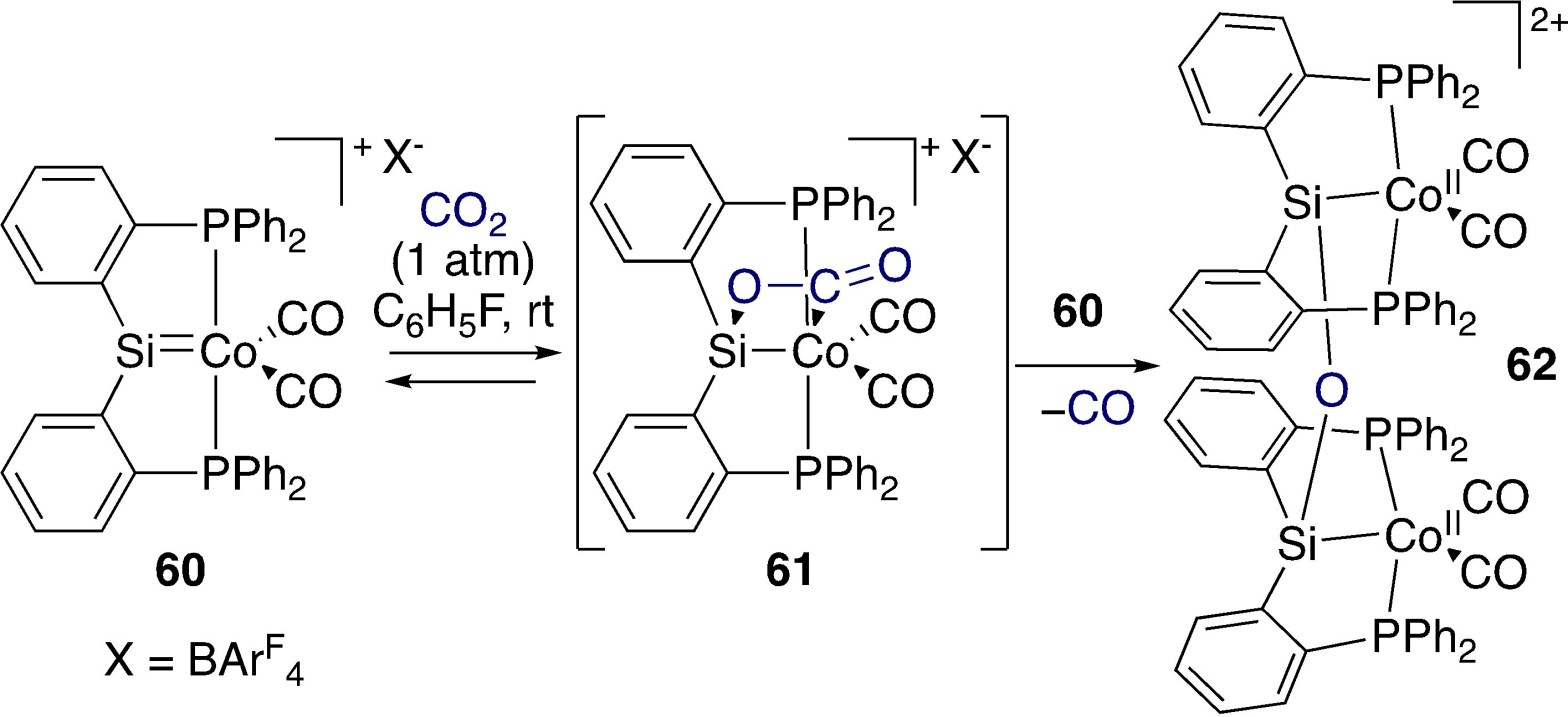
Bimetallic reduction of CO_2_ at a cobalt silylene complex.

Moving to the less reactive CO_2_, Whited et al. showed that it can be cleaved by Co‐silylene complex **60**,^[97]^ a species that had previously been shown to activate O−H bonds across the Co=Si motif.^[98]^ As shown in Scheme 20, CO_2_ is proposed to undergo a cycloaddition reaction at the polarized Co=Si bond to form intermediate **61**. This is followed by approach of another molecule of **60**, extrusion of CO, and formation of a siloxane dimer of Co(II) centres (**62**). Complex **60** also reacts with phenyl isocyanate, ethylene, and N_2_O.

**Scheme 21:**
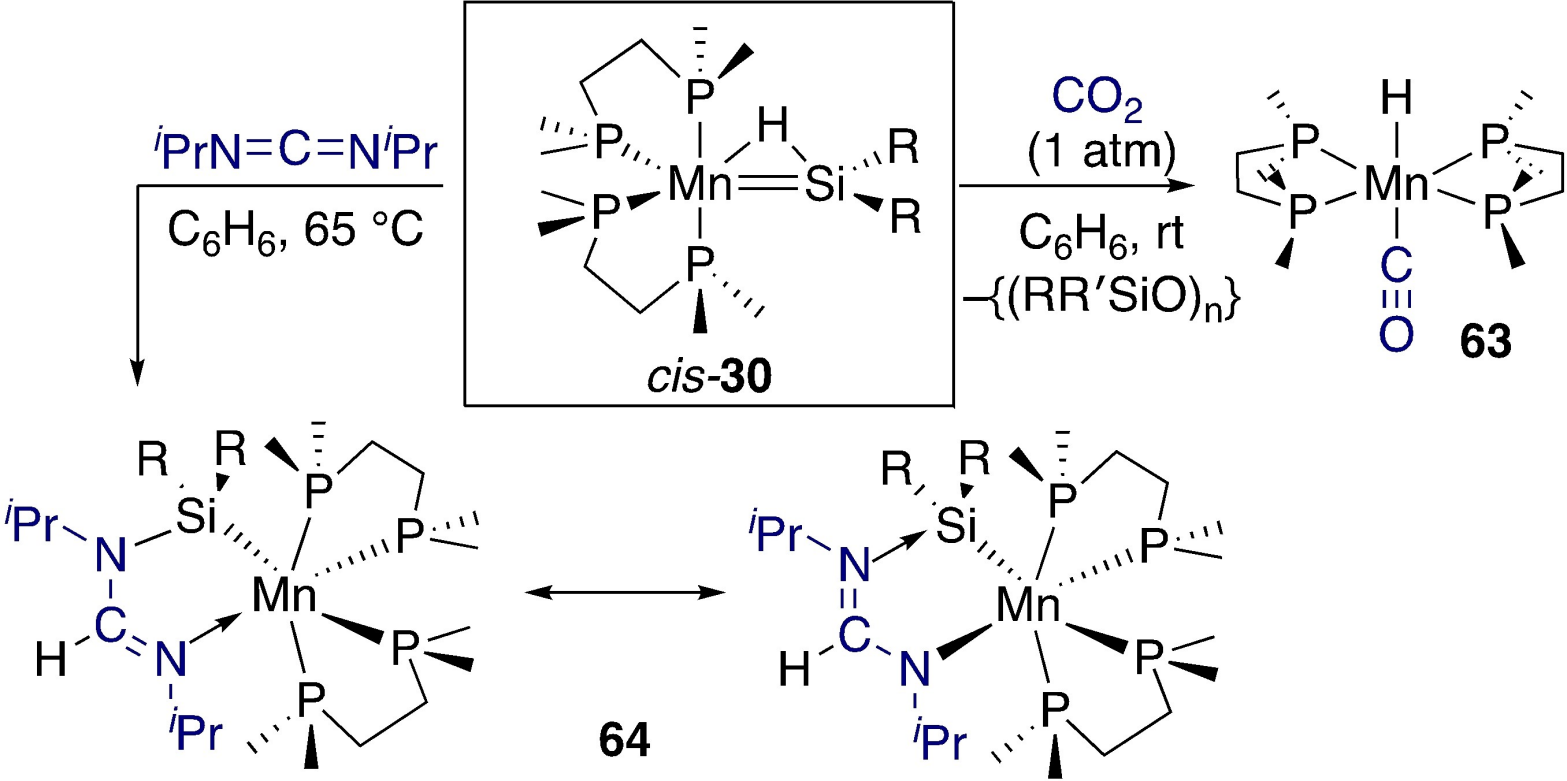
Reactivity of heteroallenes at a manganese silylene.

The Emslie group showed CO_2_ reacted with the manganese silylene hydride *cis‐* **30** and formed carbonyl complex **63** with concomitant production of polysiloxane {(RR′SiO)_n_} (Scheme 21).^[99]^ Interestingly, reaction with carbodiimide C(N^*i*^Pr)_2_ resulted in a cyclometalated product featuring a amidinylsilyl motif (**64**). Here the Mn−Si interaction remained in the final product and no C−N cleavage was observed. The authors discussed numerous potential routes for the activation of these heteroallenes, but no definitive mechanistic proposal was made.

**Scheme 22:**
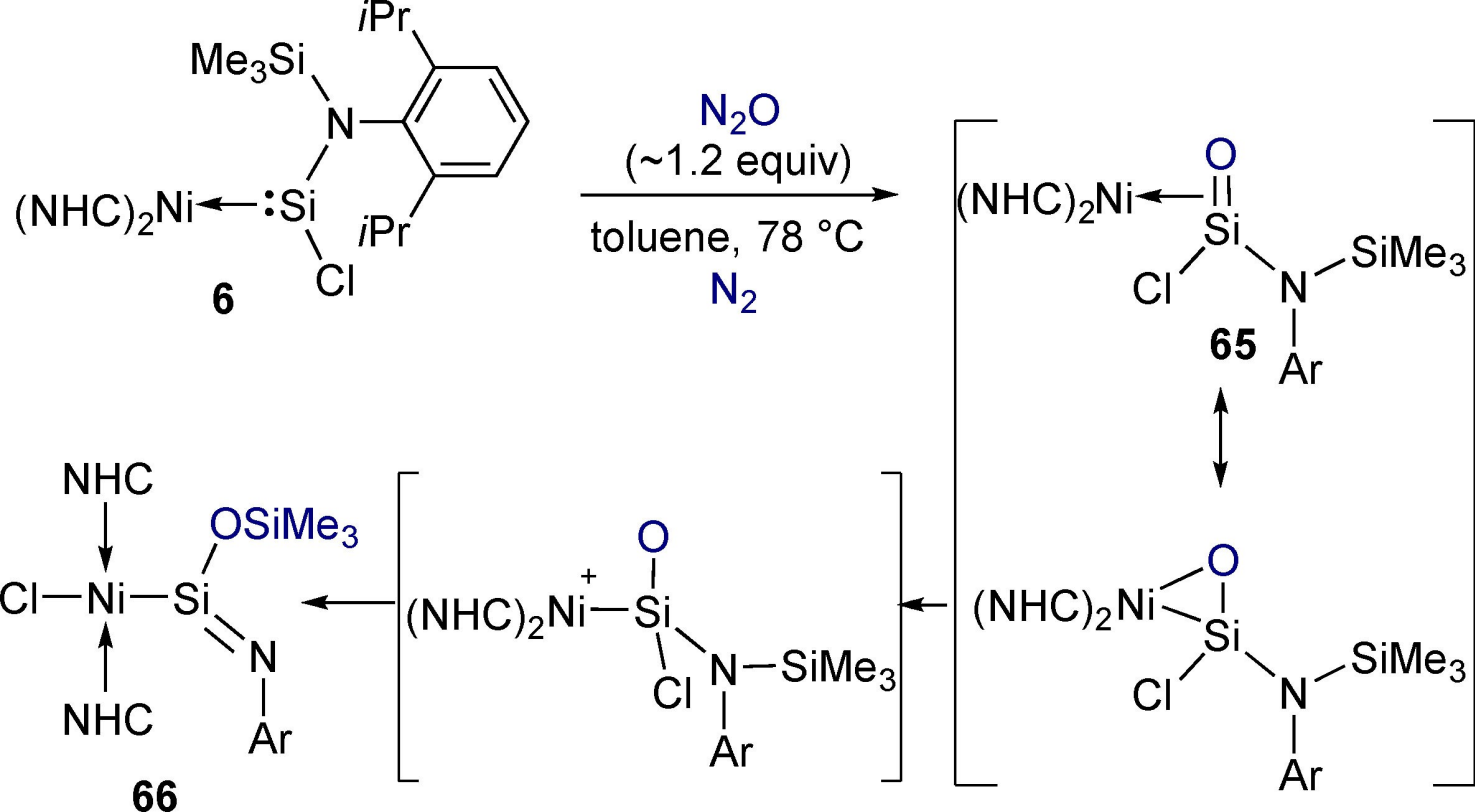
Reaction of a nickel(0) silylene with N_2_O to form a Ni(II) iminosilane via a rare metal‐stabilised silanone intermediate.

Driess and coworkers investigated the reactivity of nickel silylene complex **6** with N_2_O (Scheme 22).^[100]^ Oxidation of the Si centre by N_2_O formed an unexpected silanone π‐complex (**65**) that rearranged at very low temperature to metallo‐iminosilane complex **66**. In the final rearrangement, the nickel centre cleaves the Si−Cl bond in an example of a substrate on the original tetrylene moving to the metal centre. The Ni−Si=N bond in **66** is rare and has Si^+^−N^−^ character. The presence of a nickel‐stabilised intermediate containing a Si=O bond – a heavier analogue of a carbonyl – is interesting as such bonds are rare and tend to be highly reactive and prone to oligomerisation.^[19]^ Confirming the presence of the silanone intermediate was therefore key to this investigation. In one experiment, N_2_O was added to a sample of **6** at −78 °C and the ^29^Si NMR spectrum collected (−40 °C). The resulting ^29^Si signal at −105 ppm was consistent with previously reported base‐stabilised silanones and with the calculated chemical shift. Trapping experiments were also carried out and the reactivity of the Si=O and Si=N bonds of compounds **65** and **66** probed with a range of substrates containing H−E bonds (E=Cl, OH, B).

## 4 Summary and Outlook

It is hoped that the reactivity of transition metal/tetrylene complexes presented in the preceding sections inspires efforts to build this into innovative catalysts and to see heavier tetrylenes as more than ligands or curiosities. Stoichiometric examples of reactivity have been widely demonstrated and a whole set of fundamental elementary reactions across M=E fragments has been described. However, it still remains difficult to show reversible cleavage reactivity in the sense of oxidative addition/reductive elimination because of the thermodynamic stability of the E(IV) oxidised species, though the reactivity that has been shown is a promising step towards catalysts where a tetrylene moiety is a crucial element for reactivity. This allows for new reactions to be developed that are not (yet) feasible with more traditional transition metal catalysts.

## Conflict of interest

The authors declare no conflict of interest.

## Biographical Information

*Rosie Somerville studied chemistry in Aotearoa New Zealand at Te Herenga Waka – Victoria University of Wellington. She obtained her PhD in organometallic chemistry and homogeneous catalysis from the Institute of Chemical Research of Catalonia (ICIQ) under the supervision of Prof. Ruben Martin, then moved to the group of Jesús Campos in 2020 to carry out postdoctoral research on cooperativity between transition metals and heavier group 14 elements*.

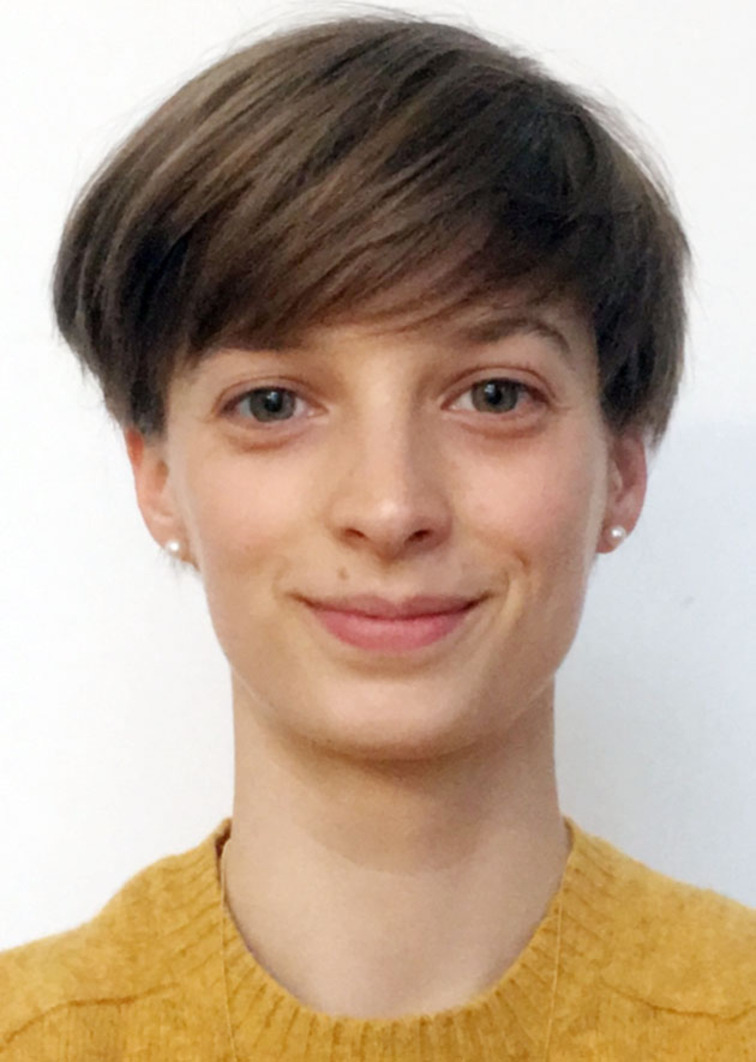

## Biographical Information

*Jesús Campos obtained his PhD in organometallic chemistry at the University of Sevilla (E. Carmona). He developed his postdoctoral research career at the universities of Yale (R. Crabtree) and Oxford (S. Aldridge). In 2016 he moved back to Sevilla as a Marie Curie fellow and a year later became CSIC permanent researcher at IIQ and was awarded an ERC Starting Grant on molecular cooperative systems. In 2020 he was appointed Fellow of the Spanish Young Academy*.